# MicroBlaze implementation of GPS/INS integrated system on Virtex-6 FPGA

**DOI:** 10.1186/s40064-015-1367-y

**Published:** 2015-10-20

**Authors:** Lokeswara Rao Bhogadi, Sasi Bhushana Rao Gottapu, VVS Reddy Konala

**Affiliations:** Department of Electronics and Communication Engineering, Andhra University College of Engineering (A), Visakhapatnam, India

**Keywords:** Field programmable gate array, Global positioning system, Inertial navigation system, Kalman filter

## Abstract

The emphasis of this paper is on MicroBlaze implementation of GPS/INS integrated system on Virtex-6 field programmable gate array (FPGA). Issues related to accuracy of position, resource usage of FPGA in terms of slices, DSP48, block random access memory, computation time, latency and power consumption are presented. An improved design of a loosely coupled GPS/INS integrated system is described in this paper. The inertial navigation solution and Kalman filter computations are provided by the MicroBlaze on Virtex-6 FPGA. The real time processed navigation solutions are updated with a rate of 100 Hz.

## Background

For autonomous navigation systems, MEMS-based inertial measurement unit (IMU) systems have proved to be highly popular and feasible (Grewal et al. 2001; Moon et al. 1998; Faulkner et al. 2002; Hegg 2002; Cao et al. 2002; Jaffe et al. 2005). These systems are cost effective, compact and light weight. The precision of the systems is not good.

Moon et al. (1998) have proposed an integrated system based on low cost IMU and GPS receiver. Faulkner et al. (2002) have proposed a digital signal processor (DSP) for developing the closely (tightly) coupled integrated system. Hegg (2002) has proposed six different power supplies for their integrated scheme. Cao et al. (2002) have proposed bridging GPS outages for tens of seconds using a low cost inertial device (Jaffe et al. 2005). Agarwal et al. (2009) have proposed an improved design and fabrication of a loosely coupled GPS/INS integrated system for compact and low power applications.

Details of the MicroBlaze implementation of the GPS/INS integrated system on Virtex-6 FPGA are presented. The emphasis is on real time issues related to accuracy of position, resource usage of FPGA in terms of slices, DSP48 and BRAM, computation time, latency and power consumption. To overcome the sensor errors and obtain accurate estimates of position and attitude, a loosely coupled integrated approach is used (Grewal et al. 2001; Moon et al. 1998).

The paper is organized as follows. “Proposed system architecture” describes about the system architecture of the proposed system. MicroBlaze implementation on Virtex-6 FPGA is presented in “Implementation with MicroBlaze”. Results and discussions are presented in “Results and discussions”. Conclusions of this work are presented in “Concluding remarks”.

## Proposed system architecture

**Fig. 1:**
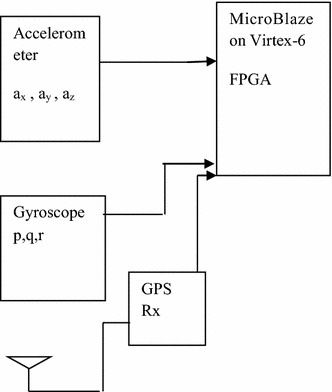
Proposed system architecture

The proposed integrated system providing the navigation system function is shown in Fig. 1. The system can be divided into four main blocks as below:

(A) INS module
(B) GPS module
(C) Sensor modelling
(D) Kalman filter module

The proposed architecture is now explained in detail.

The time history of the aircraft in the form of a state vector X is given by the flight dynamics and controls (FDC) toolbox when the initial conditions of the aircraft thrust and aerodynamics are given to FDC (Vikas Kumar 2004).

$${\text{X}} = \left[ {\upphi \; \uptheta \; \uppsi \;{\text{ p q r a}}_{{{\text{x}} }} \; {\text{a}}_{\text{y}} \; {\text{a}}_{\text{z}} \; {\text{X}} \; {\text{Y}} \; {\text{Z}} \; {\text{V}}_{\text{T}} \; \upalpha \; \upbeta } \right]^{\text{T}}$$

- ϕ θ ψ are the Euler angles in radians,
- p q r are the roll, pitch and yaw rates from

the gyroscopes in radians per second,

- a_x_ a_y_ a_z_ are the accelerations from the accelerometers in m/s^2^,
- X Y Z are the distances along the three axes in the navigation frame in meters,
- V_T_ α β are the velocity of the aircraft in m/s, the angle of attack in radians and the sideslip angle in radians, respectively.

These values can be generated by the FDC program at any time step as required. Typical update rates range from 10 to 100 ms.

### INS module

The accelerations (*a*_*x*_, *a*_*y*_ and *a*_*z*_) of the aircraft along the three body axes as read by the accelerometers, are given by the Eqs. 1–3. *U*, *V* and *W* are the velocities along the three body axes. *U*, *V* and *W* and *p*, *q*, *r* are all available as states. If the acceleration due to gravity (*g*) model is supplied as a function of location around the earth, then $\mathop {\dot{U}}\limits^{{}} ,\mathop {\dot{V}}\limits^{{}} ,\mathop {\dot{W}}\limits^{{}}$ can be calculated.

1 $$\dot{U} = a_{X} + V_{r} - W_{q} + gsin\theta$$

2 $$\dot{V} = a_{Y} - U_{r} + W_{p} - gcos\theta sin\phi$$

3 $$\dot{W} = a_{Z} + U_{q} - V_{p} - gcos\theta cos\phi$$

The earth is rotating in space at a rate *Ω* (15.0319°/h) around an axis South to North.

4 $$\varOmega = \left[ {\begin{array}{*{20}c} {\varOmega cos\lambda } \\ 0 \\ { - \varOmega sin\lambda } \\ \end{array} } \right]$$

The motion of the vehicle at a constant height above the ground will induce an additional rotation given by

5 $$\omega ' = \left[ {\begin{array}{*{20}c} {\dot{\mu }cos\lambda } \\ { - \dot{\lambda }} \\ { - \dot{\mu }sin\lambda } \\ \end{array} } \right]$$

The measured angular rates include *Ω* and $\omega^{\prime}$, we have the actual angular rates given by

6 $$\left[ {\begin{array}{*{20}c} p \\ q \\ r \\ \end{array} } \right] = \left[ {\begin{array}{*{20}c} p \\ q \\ r \\ \end{array} } \right]_{m} - {\text{DCM }}[\varOmega + \omega^{\prime} ]$$

where DCM is the direction cosine matrix or the transformation matrix, from the local earth or navigation frame to the body frame, given by Eq. 7, $\mathop {\dot{\mu }}\limits^{{}}$ is the rate of change of longitude and $\mathop {\dot{\lambda }}\limits^{{}}$ is the rate of change of latitude.

7 $$DCM = \left[ {\begin{array}{*{20}c} {cos\theta cos\psi } & {cos\theta sin\psi } & { - sin\theta } \\ {sin\theta sin\phi cos\psi - sin\psi cos\phi } & {sin\psi sin\theta sin\phi + cos\psi cos\phi } & {sin\phi cos\theta } \\ {sin\theta cos\phi cos\psi + sin\psi sin\phi } & {sin\phi sin\theta cos\phi - cos\psi sin\theta } & {cos\phi cos\theta } \\ \end{array} } \right]$$

$\mathop {\dot{U}}\limits^{{}} ,\mathop {\dot{V}}\limits^{{}} ,\mathop {\dot{W}}\limits^{{}}$ are integrated to calculate the velocity components (*U*, *V*, *W*), which are then transformed using the direction cosine matrix (Eq. 7) to give velocity along North (*V*_*N*_), velocity along East (*V*_*E*_) and downward velocity (*V*_*D*_) in the navigation frame or local earth frame.

8 $$\left[ {\begin{array}{*{20}c} {\dot{X}} \\ {\dot{Y}} \\ {\dot{Z}} \\ \end{array} } \right] = \left[ {\begin{array}{*{20}c} {V_{N} } \\ {V_{E} } \\ {V_{D} } \\ \end{array} } \right] = DCM^{T} \left[ {\begin{array}{*{20}c} U \\ V \\ W \\ \end{array} } \right]$$

*V*_*N*_, *V*_*E*_ and *V*_*D*_ are then integrated to give distances moved along the navigation axes (*X*, *Y*, *Z*) on the surface of the earth. If *λ*, *µ* and *H* denote the latitude, longitude and height of the aircraft at any instant, then the rate of change of latitude (Collison 1996; Etkin 1972) is given by

9 $$\dot{\lambda } = \frac{{V_{N} }}{{R_{e} }}$$

and rate of change of longitude is given by

10 $$\dot{\mu } = \frac{{V_{E} }}{{R_{e} cos\lambda }}$$

where *R*_*e*_ is the radius of the earth. The rate of change of altitude of the aircraft is given by

11 $$\dot{H} = - V_{D}$$

The position of the aircraft in terms of latitude, longitude and altitude can be thus calculated using Eqs. 9, 10 and 11.

From the time history, viz. ${\text{p}},{\text{ q}},{\text{ r}},{\text{ a}}_{{{\text{x}} , }} {\text{a}}_{\text{y}} ,{\text{ a}}_{\text{z}}$_,_ the INS program now takes 6 states. These act as if the program is reading directly from the gyros and accelerometers. The four Euler parameters are calculated by the program. The Euler parameters are used to calculate the Euler angles. The U, V, W are computed from the accelerations of the accelerometers.

The velocity components of the aircraft are in the body frame. To convert it to the navigation frame or local earth frame, the DCM matrix is used and V_T_ is calculated_._

**Fig. 2:**
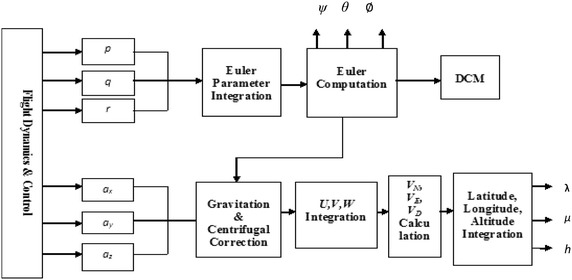
INS module

The positions *X, Y, Z* along the three axes in the local earth frame are obtained by integrating the velocity components. The latitude, longitude and height can be calculated. The fourth order Runge–Kutta method is used to carry out all the integrations. The INS module is shown in Fig. 2 (Vikas Kumar 2004).

### GPS module

The latitude, longitude and altitude of the current location are given by the GPS receiver. The GPS program converts the *X, Y, Z* given out by the FDC into latitude, longitude and altitude as would be given out by the GPS receiver. The update rate is 1 s. WGS-84 approximation is used by the GPS program. The earth is considered as an ellipse with a semi-major axis (equatorial radius) of a = 6,378,137 m, and a semi-minor axis (polar radius) of b = 6,356,752.3142 m. The distance corresponding to a 1° change in longitude (F_lon_) and latitude (F_lat_) for a specified location (latitude and height or altitude) are defined.

12 $$F_{lon} = \frac{\pi }{{180{^\circ }}}\left( {\frac{{a^{2} }}{{\sqrt {a^{2} cos^{2} \lambda + b^{2} sin^{2} \lambda } }} + h} \right)cos\lambda$$

13 $$F_{lat} = \frac{\pi }{{180{^\circ }}}\left( {\frac{{a^{2} b^{2} }}{{(a^{2} cos^{2} \lambda + b^{2} sin^{2} \lambda )^{{\frac{3}{2}}} }} + h} \right)$$

**Fig. 3:**
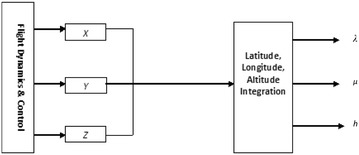
GPS module

Hence, the latitude and longitude from the previous location (λ_1_, μ_1_) are used to calculate the latitude and longitude at the current location (λ_2_, μ_2)_.

14 $$\lambda_{2} = \frac{\delta X}{{F_{lat} }} + \lambda_{1}$$

15 $$\mu_{2} = \frac{\delta Y}{{F_{lon} }} + \mu_{1} .$$

where *δX* and *δY* are the changes in position along North and East directions on the Earth, respectively. The GPS module is shown in Fig. 3 (Vikas Kumar 2004).

### Sensor modelling

Acceleration in terms of g is sent by the accelerometer to the INS in Volts. The scale factor and the bias details are available from the specification sheets of the accelerometers. As the scale factor and the bias are not fixed, errors arise in the accelerations sensed by the accelerometers (Vikas Kumar 2004). They vary stochastically and are specified in the data sheets of the accelerometers.

The corresponding scale factors and offset biases are accounted in a similar way for the gyroscope error modelling (Vikas Kumar 2004). These errors together lead to drift, which grows with time in the output (location) given by the INS and it could be up to hundreds of meters. Table 1 gives a set of values given by the specification sheets which were used in the simulation (http://www.analog.com, ADXL212, Analog Devices Inc. 2013a, DXRS652, Analog Devices Inc. 2013b; http://www.falcom.com, GPS-Receiver JP3, Falcom Wireless Communications 2013). The temperature effects and the misalignment of accelerometers and gyroscopes also cause errors. These errors have been ignored.

**Table 1:** Sensor specifications used in simulation

Quality	Value	Standard deviation
Scale factor of the accelerometer	250 mV/g	±25/3 mV/g
0 g offset of the gyroscope	2500 mV	±625/3 mV
Scale factor of the gyroscope	1.11 mV/°/s	±10/3 %
Typical turn-on drift of the gyroscope	0.12°/s	–
Random noise incorporated in the GPS	–	±20 m

### Kalman filter module

The error dynamics model given in the works of Schmidt (1978), Bar-Itzhack et al. (1988), and Grewal et al. (2001) has been used for simulation. When the nominal equations are perturbed in the local level north-pointing coordinate system that corresponds to the geographic location indicated by the INS, the error dynamics equations are obtained. The equations describing the propagation of the translatory and attitude errors describe the error behaviour of the INS. The translatory and the attitude errors are not coupled to each other. The nine state INS/GPS integration Kalman filter will then be built using the error dynamics equations. The perturbation of the position, velocity, attitude DCM, and gravity can be expressed as

16 $$\hat{r}^{n} = r^{n} + \delta r^{n}$$

17 $$\hat{v}^{n} = v^{n} + \delta v^{n}$$

18 $$\hat{C}_{b}^{n} = \left( {I - E^{n} } \right)C_{b}^{n}$$

19 $$\gamma^{\text{n}} = {\text{ g}}^{\text{n}} + \delta {\text{ g}}^{\text{n}}$$

where *r*^*n*^, *v*^*n*^ and *γ*^*n*^ denote the position, velocity and gravity vectors respectively in the navigation frame, $C_{b }^{n}$ denotes the attitude direction cosine matrix from the navigation frame to the body frame and $E^{n}$ is the skew symmetric form of the attitude errors (*ɛ*^n^)

20 $$E^{n} = \left( {{ \in }^{n} X} \right) = \left[ {\begin{array}{*{20}c} 0 & { - { \in }_{D} } & {{ \in }_{E} } \\ {{ \in }_{D} } & 0 & { - { \in }_{N} } \\ { - { \in }_{E} } & { - { \in }_{N} } & 0 \\ \end{array} } \right]$$

and ^ and δ denote computed values and errors, respectively.

The linear position error dynamics can be denoted by the perturbing Eqs. 9–11, which are the dynamics equations for the geodetic positions. Since the position dynamics equations are functions of position and velocity, the position error dynamics equations are obtained using partial derivatives (Shin 2001).

21 $$\delta v\delta \hat{r}^{n} = F_{rr} \delta r^{n} + F_{rv}^{n}$$

where

$$F_{rr} = \left( {\begin{array}{*{20}c} {\frac{{\partial \dot{\lambda }}}{\partial \lambda }} & {\frac{{\partial \dot{\lambda }}}{\partial \mu }} & {\frac{{\partial \dot{\lambda }}}{\partial h}} \\ {\frac{{\partial \dot{\mu }}}{\partial \lambda }} & {\frac{{\partial \dot{\mu }}}{\partial \mu }} & {\frac{{\partial \dot{\mu }}}{\partial h}} \\ {\frac{{\partial \dot{h}}}{\partial \lambda }} & {\frac{{\partial \dot{h}}}{\partial \mu }} & {\frac{{\partial \dot{h}}}{\partial h}} \\ \end{array} } \right) = \left( {\begin{array}{*{20}c} 0 & 0 & {\frac{{ - V_{N} }}{{(R_{e} + h)^{2} }}} \\ {\frac{{V_{E} sin\lambda }}{{\left( {R_{e} + h} \right)cos^{2} \lambda }}} & 0 & {\frac{{ - V_{E} }}{{(R_{E} + h)^{2} cos\lambda }}} \\ 0 & 0 & 0 \\ \end{array} } \right)$$

$$F_{rv} = \left( {\begin{array}{*{20}c} {\frac{{\partial \dot{\lambda }}}{{\partial V_{N} }}} & {\frac{{\partial \dot{\lambda }}}{{\partial V_{E} }}} & {\frac{{\partial \dot{\lambda }}}{{\partial V_{D} }}} \\ {\frac{{\partial \dot{\mu }}}{{\partial V_{N} }}} & {\frac{{\partial \dot{\mu }}}{{\partial V_{E} }}} & {\frac{{\partial \dot{\mu }}}{{\partial V_{D} }}} \\ {\frac{{\partial \dot{h}}}{{\partial V_{N} }}} & {\frac{{\partial \dot{h}}}{{\partial V_{E} }}} & {\frac{{\partial \dot{h}}}{{\partial V_{D} }}} \\ \end{array} } \right) = \left( {\begin{array}{*{20}c} {\frac{1}{{R_{e} + h}}} & 0 & 0 \\ 0 & {\frac{1}{{\left( {R_{e} + h} \right)cos\lambda }}} & 0 \\ 0 & 0 & { - 1} \\ \end{array} } \right)$$

and R_e_ is the radius of the earth and is considered a constant. The velocity dynamics equation is expressed as

22 $$\hat{\dot{v}}^{n} = \hat{C}_{b}^{n} f^{b} - \left( {2\varOmega + \omega^{\prime} } \right) \times \hat{v}^{n} + \gamma^{n}$$

where $f^{b}$ is the acceleration of the aircraft in the body frame. The gravitation vector in the navigation frame g^n^, can be approximated by the normal gravity $\left( {0\, 0 \, \gamma } \right)^{\text{T}}$, and *γ* varies with altitude. Assuming a spherical model, *γ* is given by

23 $$\gamma = \gamma_{0} \left( {\frac{{R_{e} }}{{R_{e} + h}}} \right)^{2} ,$$

where $\gamma_{0}$ is the normal gravity at *h* = 0. On perturbating Eq. 22 the velocity error dynamics equation can be obtained as (Shin 2001)

24 $$\delta \dot{v}^{n} = F_{vr} \delta r^{n} + F_{vv} \delta v^{n} + \left( {f^{n} X} \right){ \in }^{n} + C_{b}^{n} \delta f^{b}$$

where

$$F_{vr} = \left( {\begin{array}{*{20}c} { - 2V_{E} \varOmega cos\lambda - \frac{{V_{E}^{2} }}{{\left( {R_{e} + h} \right)cos^{2} \lambda }}} & 0 & {\frac{{ - V_{N} V_{D} }}{{(R_{e} + h)^{2} }} + \frac{{V_{E}^{2} tan\lambda }}{{(R_{e} + h)^{2} }} } \\ {2\varOmega \left( {V_{N} cos\lambda - V_{D} sin\lambda } \right) + \frac{{V_{E} V_{N} }}{{\left( {R_{e} + h} \right)cos^{2} \lambda }}} & 0 & {\frac{{V_{E} V_{D} }}{{(R_{e} + h)^{2} }} - \frac{{V_{N} V_{E} tan\lambda }}{{(R_{e} + h)^{2} }} } \\ {2V_{E} \varOmega sin\lambda } & 0 & {\frac{{V_{E}^{2} + V_{N}^{2} }}{{(R_{e} + h)^{2} }} - \frac{2\gamma }{{\left( {R_{e} + h} \right)}}} \\ \end{array} } \right)$$

$$F_{vv} = \left( {\begin{array}{*{20}c} {\frac{{V_{D} }}{{R_{e} + h}}} & { - 2\varOmega sin\lambda - 2\frac{{V_{E} tan\lambda }}{{R_{e} + h}}} & {\frac{{V_{N} }}{{R_{e} + h}}} \\ {2\varOmega sin\lambda + \frac{{V_{E} tan\lambda }}{{R_{e} + h}}} & {\frac{{V_{D} + V_{N} tan\lambda }}{{R_{e} + h}}} & {2\varOmega cos\lambda + \frac{{V_{E} }}{{R_{e} + h}}} \\ { - 2\frac{{V_{N} }}{{R_{e} + h}}} & { - 2\varOmega cos\lambda - 2\frac{{V_{E} }}{{R_{e} + h}}} & 0 \\ \end{array} } \right)$$

and δf^b^ is the perturbation in the acceleration vector in the body frame.

The attitude error dynamics equation (Shin 2001) can be written as

25 $$\dot{\varepsilon }^{n} = F_{er} \delta r^{n} + F_{ev} \delta v^{n} + \left( {(\varOmega + \omega ')X^{ } } \right){ \in }^{n} - C_{b}^{n} \delta \omega_{ib}^{b}$$

where

$$F_{er} = \left( {\begin{array}{*{20}c} { - \varOmega sin\lambda } & 0 & {\frac{{ - V_{E} }}{{(R_{e} + h)^{2} }}} \\ 0 & 0 & {\frac{{V_{N} }}{{(R_{e} + h)^{2} }}} \\ { - \varOmega cos\lambda - \frac{{V_{E} }}{{\left( {R_{e} + h} \right)cos^{2} \lambda }}} & 0 & {\frac{{V_{E} tan\lambda }}{{(R_{e} + h)^{2} }}} \\ \end{array} } \right)$$

$$F_{ev} = \left( {\begin{array}{*{20}c} 0 & {\frac{1}{{R_{e} + h}}} & 0 \\ {\frac{ - 1}{{R_{e} + h}}} & 0 & 0 \\ 0 & {\frac{ - tan\lambda }{{R_{e} + h}}} & 0 \\ \end{array} } \right)$$

and $\delta \omega_{ib}^{b}$ is the perturbation in the angular rate vector between the inertial frame and the body frame.

A state space model can be constructed by augmenting the Eqs. 21, 22 and 25 as follows

26 $$\dot{X} = FX + Gu$$

where *F* is the dynamics matrix, $X$ is the state vector, *G* is a design matrix, *u* is the forcing vector function (Shin 2001):

$$F = \left( {\begin{array}{*{20}c} {F_{rv} } & {F_{rv} } & 0 \\ {F_{vr} } & {F_{vv} } & {(f^{b} X)} \\ {F_{er} } & {F_{ev} } & { - (\left( {\varOmega + \omega^{\prime} } \right)X} \\ \end{array} } \right) \quad {\text{x}} = \left( {\begin{array}{*{20}c} {\delta r^{n} } \\ {\delta v^{n} } \\ {{ \in }^{n} } \\ \end{array} } \right)$$

$$G = \left( {\begin{array}{*{20}c} 0 & 0 \\ {C_{b}^{n} } & 0 \\ 0 & { - C_{b}^{n} } \\ \end{array} } \right) \quad u = \left( {\begin{array}{*{20}c} {\delta f^{b} } \\ {\delta \omega_{ib}^{b} } \\ \end{array} } \right)$$

The elements of *u* are white noise whose covariance matrix is given by

27 $$E[u\left( t \right)u(t)^{T} = Q\left( t \right)\delta \left( {t - T} \right)$$

where the operator δ denotes the Dirac delta function whose unit is 1/time (Shin 2001). Q is called the spectral density matrix and has the form

28 $$Q = diag\left( {\sigma_{ax}^{2 }\; \sigma_{ay}^{2} \;\sigma_{az}^{2} \;\sigma_{\omega x }^{2} \;\sigma_{wx}^{2}\; \sigma_{wz}^{2} } \right)$$

where *ϭ*_*a*_ and *ϭ*_*ɷ*_ are the standard deviations of the accelerometers and gyroscopes, respectively.

The Eq. 26 in discrete time form is given by

29 $$X_{k + 1} = \varPhi_{k} X_{k} + w_{k}$$

where $\varPhi_{k}$ is the state transition matrix and *w*_*k*_ is the driven response at *t*_*k*+*1*_ due to the presence of input white noise during time interval (*t*_*k*_, *t*_*k*+*1*_) (Brown and Hwang 1992). For the implementation of the INS, because the time interval Δ*t* = *t*_*k*+*1*_ − *t*_*k*_ is very small, the state transition matrix can be numerically approximate as

30 $$\varPhi_{k} = exp\left[ {F\Delta t} \right] \approx I + F\Delta t$$

The covariance matrix associated with *w*_*k*_ is

31 $$Q_{k} = E\left[ {w_{k} w_{k}^{T} } \right] \approx \varPhi_{K} GQG^{T} \varPhi_{k}^{T} \Delta t$$

If the norm of $Q_{k}$ is larger than the real one, the Kalman filter trusts the measurements more than the system, thus making the estimates noisy due to free passage of measurement noise (Shin 2001). However, there is no time lag. If the norm of $Q_{k}$ is less than one, the time lag exists. When the norm of $Q_{k}$ is much smaller than the real one, the filter diverges, which may result in numerical instabilities. Hence, for low inertial systems, $Q_{k}$ must be selected pessimistically so that the trajectory follows that of the GPS. The elements corresponding to *δf*_*z*_ should be large enough so that they can account for the uncertainties in gravity as well as sensor imperfection.

The process noise, *w*_*k*_ and the measurement noise, *v*_*k*_ are uncorrelated, hence their covariance is 0. The covariance matrix for *v*_*k*_ is given by

32 $$E\left[ {v_{k} v_{k}^{T} } \right] = R_{k}$$

The Kalman filter is then implemented. The position from GPS is considered as measurements. The formulation of the measurement of the measurement equation can be written as

33 $$z_{k} = r_{INS}^{n} - r_{GPS}^{n} = \left( {\begin{array}{*{20}c} {\lambda_{INS} - \lambda_{GPS} } \\ {\mu_{INS} - \mu_{GPS} } \\ {h_{INS} - h_{GPS} } \\ \end{array} } \right) \quad H_{k} = \left\langle {I_{3x3} |0_{3x3} |0_{3x3} } \right\rangle$$

Since *λ* and *μ* are in radians and hence very small, they cause numerical instabilities in calculating the Kalman gain ***K***_***k***_. Hence, the first two rows are multiplied by (*R*_*e*_ +*h*) and (*R*_*e*_ +*h*)*cosλ*, respectively (Shin 2001). The measurement equation now takes the form:

$$z_{k} = \left( {\begin{array}{*{20}c} {\left( {R_{e} + h} \right)(\lambda_{INS} - \lambda_{GPS} )} \\ {\left( {R_{e} + h} \right)cos\lambda (\mu_{INS} - \mu_{GPS} )} \\ {h_{INS} - h_{GPS} } \\ \end{array} } \right)$$

34 $$H_{k} = \left\langle {\begin{array}{*{20}c} {(R_{e} + h)} & 0 & 0 \\ 0 & {\left( {R_{e} + h} \right)cos\lambda } & 0 \\ 0 & 0 & 1 \\ \end{array} \bigg| {0_{3x3} } \bigg|0_{3x3} } \right\rangle$$

and the following measurement noise matrix has been used

35 $$R_{k} = diag(\sigma_{\lambda }^{2} \;\sigma_{\mu }^{2}\; \sigma_{h}^{2} )$$

which can be obtained from GPS processing. In our simulation, we have taken the error sphere of the GPS to have a radius of 20 m. Hence $\sigma_{\lambda } = \sigma_{\mu } = \sigma_{h} = 20{\text{m}}$.

**Fig. 4:**
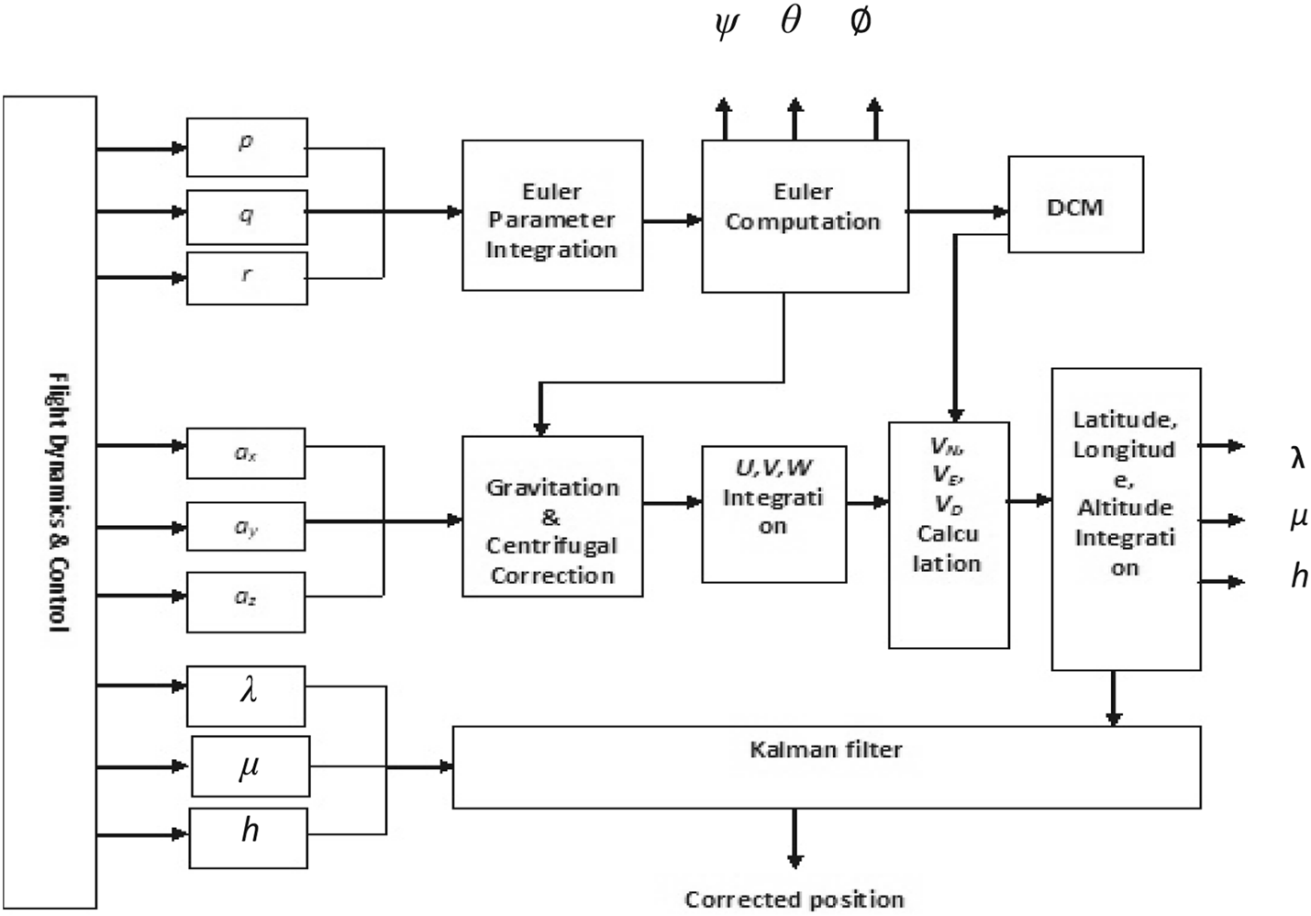
Kalman filter module

The Kalman filter module is shown in Fig. 4 (Vikas Kumar 2004).

The KF is a very effective stochastic estimator for a large number of problems, be it in computer graphics or in navigation. It is an optimal combination, in terms of minimization of variance, between the prediction of parameters from a previous time instant and external observations at a present time instant.

The MicroBlaze implementation of the GPS/INS integrated system on Virtex-6 FPGA is discussed in detail in the next section.

## Implementation with MicroBlaze

This section describes the implementation of the above described algorithm. The microprocessors available for use in Xilinx FPGAs with Xilinx Embedded Development Kit (EDK) software tools can be broken down into two broad categories. There are soft-core microprocessors (MicroBlaze) and the hard-core embedded microprocessor (Power PC). The soft-core MicroBlaze microprocessor can be used in most of the Spartan-II, Spartan-3 and Virtex FPGA families. The hard-core embedded microprocessor is an IBM PowerPC 405 processor, which is only available in the Virtex-II Pro and Virtex-4 FX FPGA’s.

The MicroBlaze is a virtual microprocessor that is built by combining blocks of code called cores inside a Xilinx FPGA. The beauty to this approach is that as much microprocessor as needed can be developed. The project can be tailored to specific needs (i.e.: Flash, UART, General Purpose Input/output peripherals and etc.).

The MicroBlaze processor is a 32-bit Harvard Reduced Instruction Set Computer (RISC) architecture optimized for implementation in Xilinx FPGAs with separate 32-bit instruction and data buses running at full speed to execute programs and access data from both on-chip and external memory at the same time. The backbone of the architecture is a single-issue, 3-stage pipeline with 32 general-purpose registers (does not have any address registers like the Motorola 68000 Processor), an arithmetic logic unit (ALU), a shift unit, and two levels of interrupt. This basic design can then be configured with more advanced features to tailor to the exact needs of the target embedded application such as: barrel shifter, divider, multiplier, single precision floating-point unit (FPU), instruction and data caches, exception handling, debug logic, fast simplex link (FSL) interfaces and others. This flexibility allows the user to balance the required performance of the target application against the logic area cost of the soft processor.

The MicroBlaze pipeline is a parallel pipeline, divided into three stages: fetch, decode, and execute. In general, each stage takes one clock cycle to complete. Consequently, it takes three clock cycles (ignoring delays or stalls) for the instruction to complete. Each stage is active on each clock cycle so three instructions can be executed simultaneously, one at each of the three pipeline stages. MicroBlaze implements an instruction prefetch buffer that reduces the impact of multi-cycle instruction memory latency. While the pipeline is stalled by a multi-cycle instruction in the execution stage the instruction prefetch buffer continues to load sequential instructions. Once the pipeline resumes execution the fetch stage can load new instructions directly from the instruction prefetch buffer rather than having to wait for the instruction memory access to complete. The instruction prefetch buffer is part of the backbone of the MicroBlaze architecture and is not the same thing as the optional instruction cache.

The MicroBlaze core is organized as a Harvard architecture with separate bus interface units for data accesses and instruction accesses. MicroBlaze does not separate between data accesses to I/O and memory (i.e. it uses memory mapped I/O). The processor has up to three interfaces for memory accesses: local memory bus (LMB), IBM’s on-chip peripheral bus (OPB), and Xilinx cache link (XCL). The LMB provides single-cycle access to on-chip dual-port block RAM (BRAM). The OPB interface provides a connection to both on-chip and off-chip peripherals and memory. The cache link interface is intended for use with specialized external memory controllers. MicroBlaze also supports up to 8 FSL ports, each with one master and one slave FSL interface. The FSL is a simple, yet powerful, point-to-point interface that connects user developed custom hardware accelerators (co-processors) to the MicroBlaze processor pipeline to accelerate time-critical algorithms.

All MicroBlaze instructions are 32 bits wide and are defined as either type A or type B. Type A instructions have up to two source register operands and one destination register operand. Type B instructions have one source register and a 16-bit immediate operand. Type B instructions have a single destination register operand. Instructions are provided in the following functional categories: arithmetic, logical, branch, load/store, and special. MicroBlaze is a load/store type of processor meaning that it can only load/store data from/to memory. It cannot do any operations on data in memory directly; instead the data in memory must be brought inside the MicroBlaze processor and placed into the general-purpose registers to do any operations. Both instruction and data interfaces of MicroBlaze are 32 bit wide and use Big-Endian, bit-reversed format to represent data (order of bits: bit 0 bit 1 … bit 30 bit 31 with bit 0 the MSB and bit 31 the LSB). MicroBlaze supports word (32 bits), half-word (16 bits), and byte accesses to data memory. Data accesses must be aligned (i.e. word accesses must be on word boundaries, half-word on half-word boundaries), unless the processor is configured to support unaligned exceptions. All instruction accesses must be word aligned.

The stack convention used in MicroBlaze starts from a higher memory location and grows downward to lower memory locations when items are pushed onto a stack with a function call. Items are popped off the stack the reverse order they were put on; item at the lowest memory location of the stack goes first and etc.

The MicroBlaze processor also has special purpose registers such as: program counter (PC) can read it but cannot write to it, machine status register (MSR) to indicate the status of the processor such as indicating arithmetic carry, divide by zero error, a FSL error and enabling/disabling interrupts to name a few. An exception address register (EAR) that stores the full load/store address that caused the exception. An exception status register (ESR) that indicates what kind of exception occurred. A floating point status register (FSR) to indicate things such as invalid operation divide by zero error, overflow, underflow and denormalized operand error of a floating point operation.

MicroBlaze also supports reset, interrupt, user exception, break and hardware exceptions. For interrupts, MicroBlaze supports only one external interrupt source (connecting to the interrupt input port). If multiple interrupts are needed, an interrupt controller must be used to handle multiple interrupt requests to MicroBlaze. An interrupt controller is available for use with the Xilinx EDK software tools. The processor will only react to interrupts if the interrupt enable (IE) bit in the MSR is set to 1. On an interrupt the instruction in the execution stage will complete, while the instruction in the decode stage is replaced by a branch to the interrupt vector (address 0 × 10). The interrupt return address (the PC associated with the instruction in the decode stage at the time of the interrupt) is automatically loaded into general-purpose register R14. In addition, the processor also disables future interrupts by clearing the IE bit in the MSR. The IE bit is automatically set again when executing the RTID instruction.

Writing software to control the MicroBlaze processor must be done in C/C++ language. Using C/C++ is the preferred method by most people and is the format that the Xilinx EDK software tools expect. The EDK tools have built in C/C++ compilers to generate the necessary machine code for the MicroBlaze processor.

The MicroBlaze processor is useless by itself without some type of peripheral devices to connect to and EDK comes with a large number of commonly used peripherals.

**Fig. 5:**
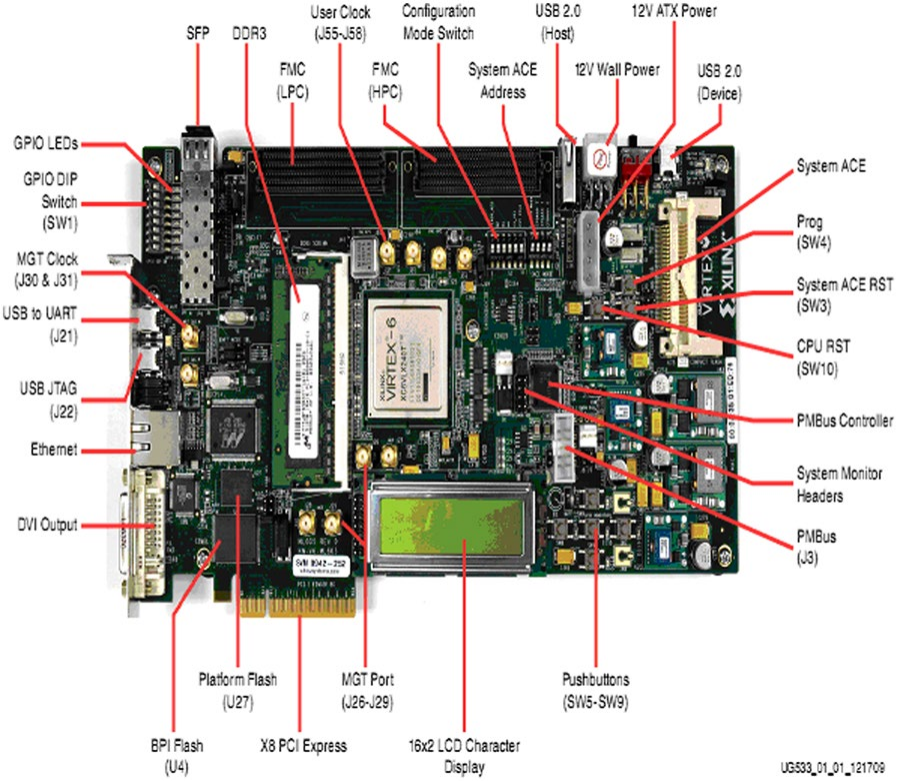
ML605 evaluation board

The GPS/INS integration with Kalman filter implemented on Virtex-6 XC6VLX240T-1FFG1156 FPGA reconfigurable hardware is shown in Fig. 5 (http://www.xilinx.com, Virtex-6 FPGA and Xilinx 2013). The choice of reconfigurable hardware is based on the envisaged applications of this GPS/INS integration system in military and high speed avionics. The ML605 evaluation board from Xilinx is used as target hardware for hardware level verification.

**Fig. 6:**
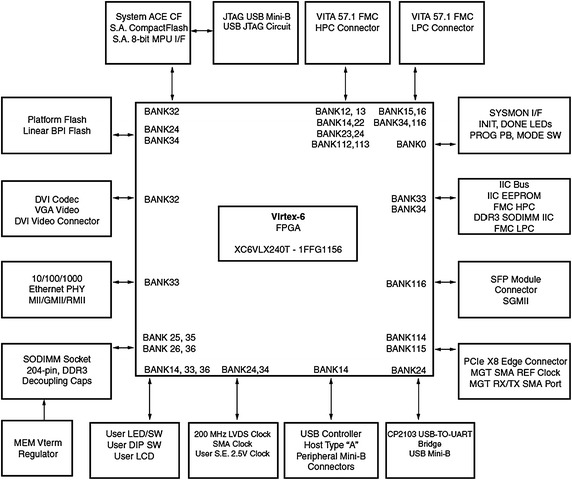
Block diagram of ML605 evaluation board

The block diagram of the ML 605 evaluation board is shown in Fig. 6 (http://www.xilinx.com, Virtex6FPGA and Xilinx 2013).

**Fig. 7:**
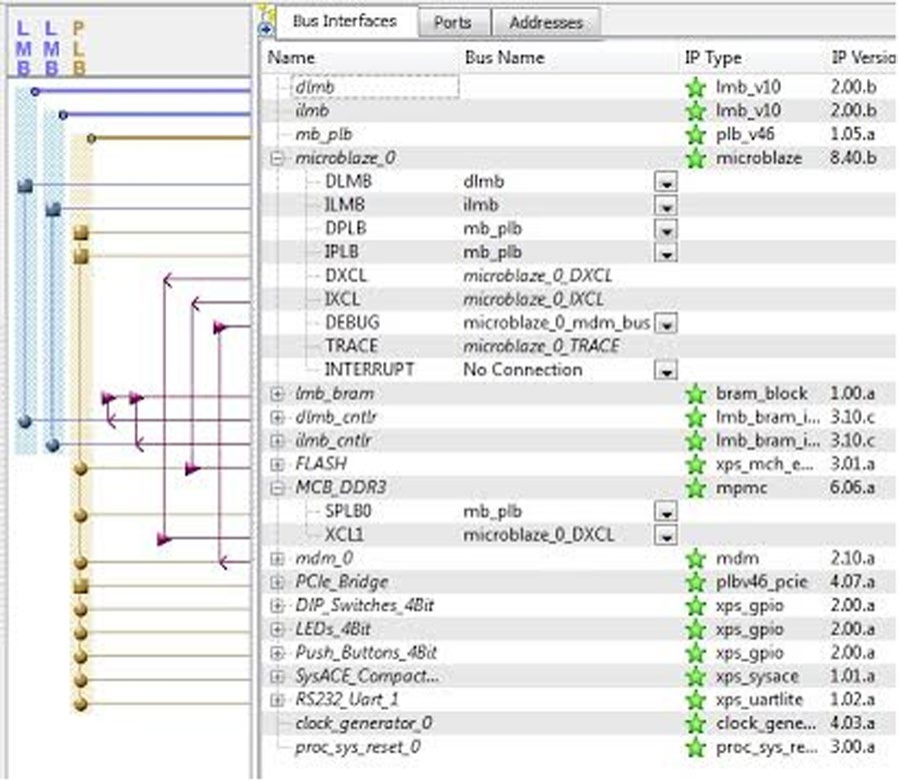
Hardware plat form with MicroBlaze and peripherals

The hardware software co-design flow with Xilinx’s MicroBlaze softprocessor core is adopted here. The INS data server, GPS data server are implemented as hardware intellectual property (IP) cores which provide the data samples to the MicroBlaze softprocessor. The INS and GPS data servers are the same as the INS and GPS modules that are already described in the previous sections. The Kalman filtering algorithm is implemented in C and ported to MicroBlaze along with its board support files. The IEEE 754 single precession floating point unit (FPU) core is added to MicroBlaze as a peripheral unit. The data taken for processing through Kalman filter is of floating point numbers with 6 fractional bits. The bit growth through Kalman filter processing is easily taken care for these fractional bits through single precision floating point numbers. Usage of double precision floating numbers is always preferable but at the cost of increased resources in FPGA. The entire processor local bus (PLB) based hardware system configuration using Xilinx platform studio (XPS) is shown in Fig. 7 (http://www.xilinx.com, Virtex6FPGA and Xilinx 2013).

The USB-UART available on board is used for reading the algorithm inputs and outputs to personal computer (PC) for further analysis. The onboard JTAG debugger is used for configuration and runtime debugging. MATLAB is used for analyzing the logged data and results are presented in next section.

## Results and discussions

**Fig. 8:**
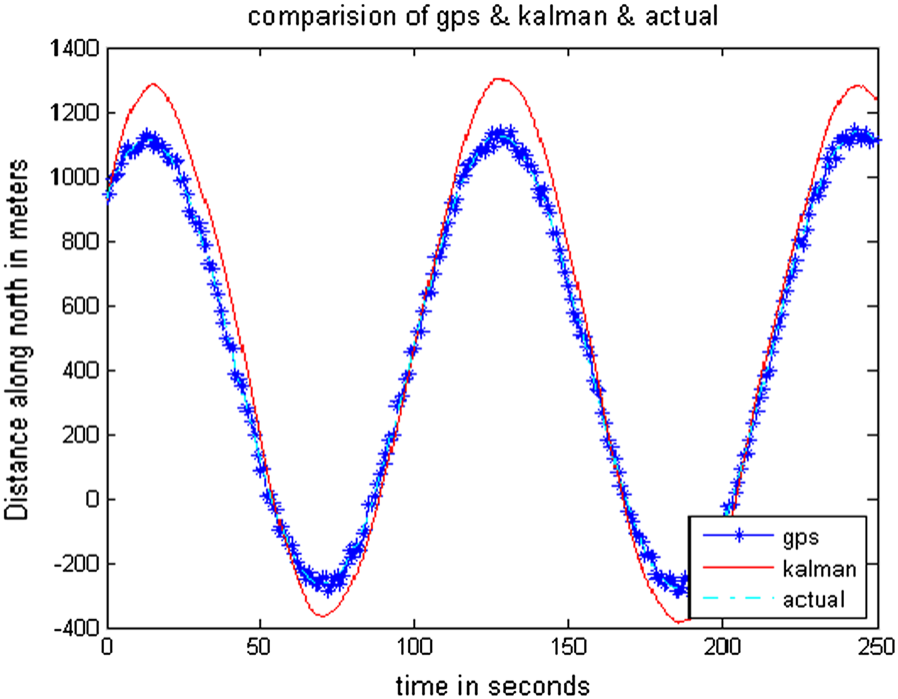
Distance along North as given by the Kalman filter

**Fig. 9:**
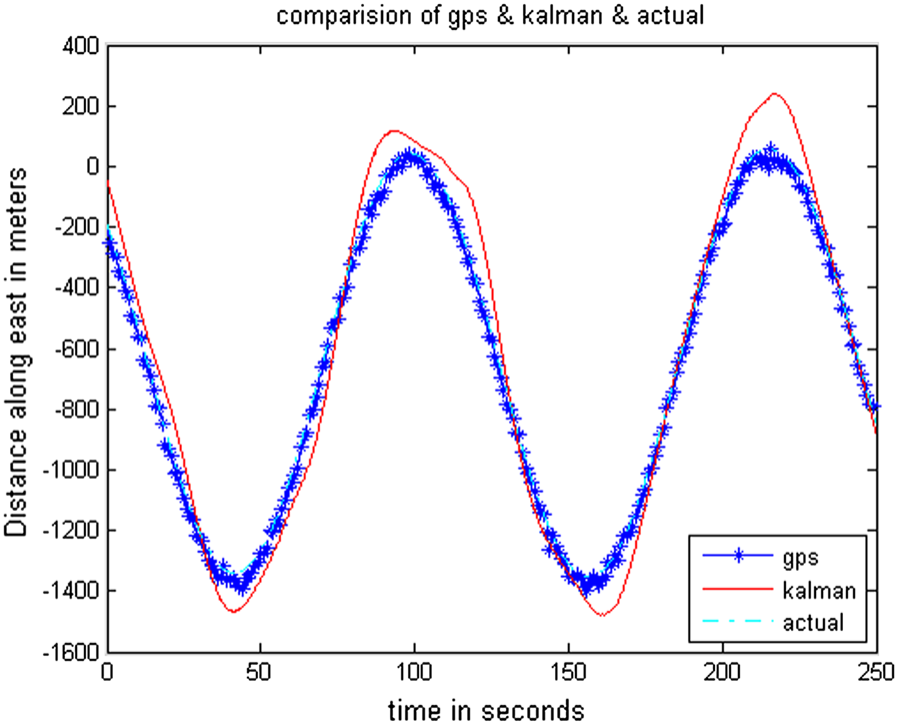
Distance along East as given by the Kalman filter

**Fig. 10:**
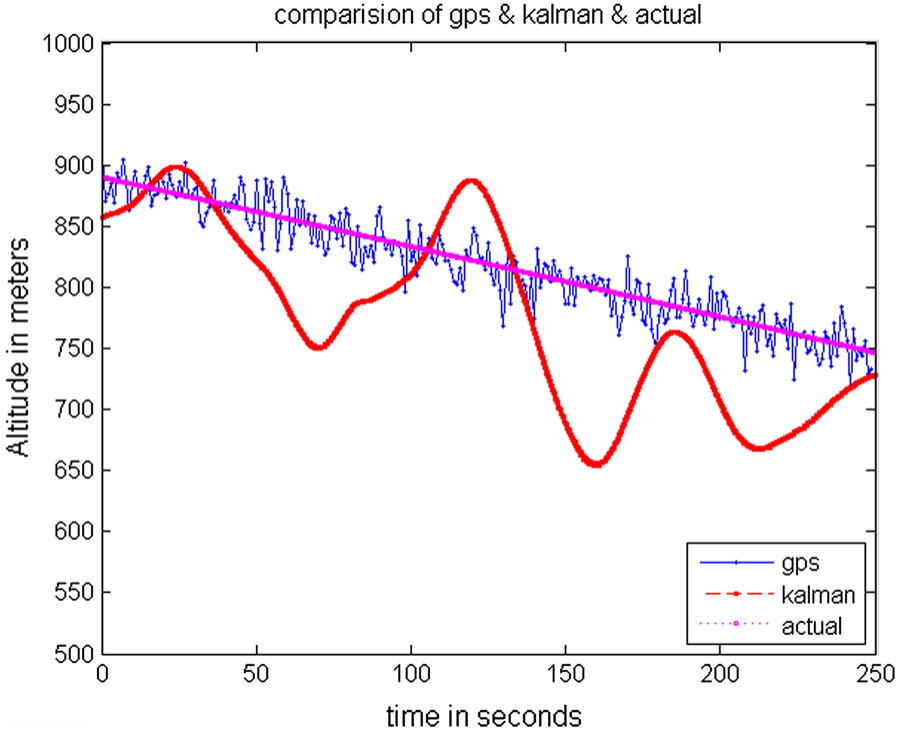
Altitude as given by the Kalman filter

**Fig. 11:**
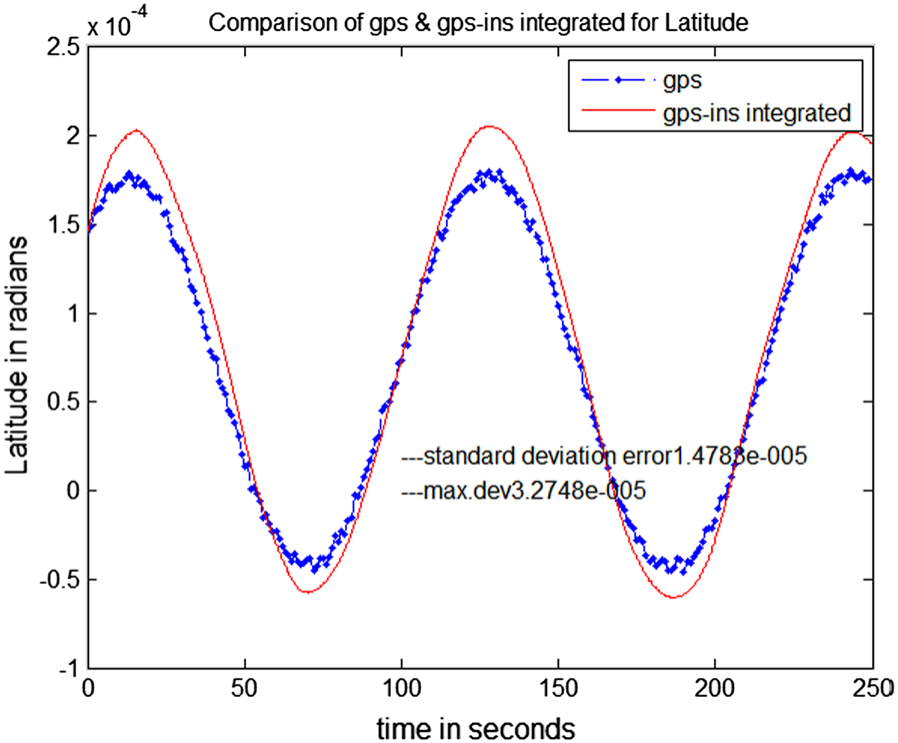
Virtex-6 FPGA (hardware) output. Latitude versus time

**Fig. 12:**
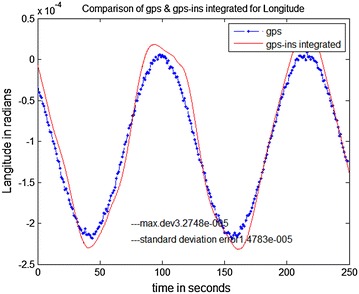
Virtex-6 FPGA (hardware) output. Longitude versus time

**Fig. 13:**
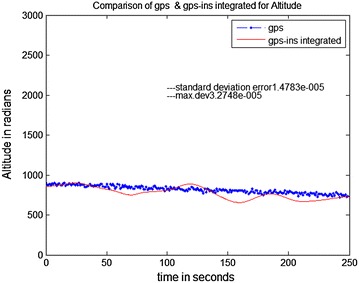
Virtex-6 FPGA (hardware) output. Altitude versus time

The data stored on the hard drive is input for the system. The program is run just as if the collection (of data) was taking place in real-time. Data is taken from FDC for 10 ms and analysis is done through MATLAB. The trajectories obtained from the FDC in MATLAB give the simulated sensor data. The stored Aircraft states were used to simulate sensors. The prediction accuracy of INS is analyzed by comparing it with a true trajectory generated using MATLAB. Various errors in inertial sensors and the GPS are included in the simulation results. Same sensor outputs were given to the present MicroBlaze implementation on Vitex-6 FPGA and the results were compared. The output waveforms obtained from the GPS/INS integrated system, the GPS, and the actual trajectory are shown in Figs. 8, 9 and 10. The plots for latitude, longitude, and altitude obtained directly from the hardware (Virtex-6 FPGA output) are shown in Figs. 11, 12 and 13. They show good agreement with the actual trajectory.

The resource utilization report of the developed system on Virtex-6 FPGA is given in Table 2. As the area consuming algorithm blocks are implemented on MicroBlaze and performance demanding blocks are implemented on FPGA fabrics, the implemented system demonstrates optimal area occupancy and high speed implementation.

**Table 2:** Resource utilization of Virtex-6 FPGA

Resource	Total available	Used	Utilization (%)
Slice registers	3,01,440	7101	2
Slice LUTs	1,50,720	7925	5
BRAM	416	70	16
DSP48	768	5	1

The maximum clock speed achieved by MicroBlaze softprocessor is 130 MHz. The present system is tested at 88 MHz clock speed. The FPGA running for the total integrated system is profiled for speed and latencies by running it for 900 s of data.

The Kalman filter iterations are presently being computed at 10 ms interval. However the implemented logic is able to achieve the latency of 0.9 ms for every iteration. Hence the present system can integrate with INS system with an update rate of 1 ms.

## Concluding remarks

The paper discussed a better approach to fuse the data from the GPS and INS using Kalman filter. The position accuracy of the GPS/INS system is comparable to that of the GPS receiver over a span of 250 s.

The implemented system with hardware software co-design approach occupies only 5 % of slices and 1 % of DSP48 resources, with maximum achievable clock speed of 130 MHz. The latency for one iteration of Kalman filter is less than 1 ms, hence suitable for integrating with high speed INS units. The Virtex-6 FPGA consumes 4285 mW of power. The Kalman filter implemented on Virtex-6 FPGA shows promising results by using reconfigurable hardware software co design approach for future GPS/INS integrated navigation systems. The power consumption is calculated for the operating clock rate of 100 MHz.
